# Micronucleated Erythrocytes in Peripheral Blood from Neonate Rats Exposed by Breastfeeding to Cyclophosphamide, Colchicine, or Cytosine-Arabinoside

**DOI:** 10.1155/2016/9161648

**Published:** 2016-11-28

**Authors:** Belinda C. Gómez-Meda, Luis R. Bañales-Martínez, Ana L. Zamora-Perez, María de Lourdes Lemus-Varela, Xóchitl Trujillo, María G. Sánchez-Parada, Blanca M. Torres-Mendoza, Juan Armendáriz-Borunda, Guillermo M. Zúñiga-González

**Affiliations:** ^1^Instituto de Biología Molecular en Medicina y Terapia Génica, Departamento de Biología Molecular y Genómica, Centro Universitario de Ciencias de la Salud, Universidad de Guadalajara, Guadalajara, JAL, Mexico; ^2^Departamento de Neonatología, Hospital de Pediatría, Centro Médico Nacional de Occidente, Instituto Mexicano del Seguro Social, Guadalajara, JAL, Mexico; ^3^Instituto de Investigación en Odontología, Departamento de Clínicas Odontológicas Integrales, Centro Universitario de Ciencias de la Salud, Universidad de Guadalajara, Guadalajara, JAL, Mexico; ^4^Centro Universitario de Investigaciones Biomédicas, Universidad de Colima, Colima, COL, Mexico; ^5^Departamento de Ciencias de la Salud, Centro Universitario de Tonalá, Universidad de Guadalajara, Tonalá, JAL, Mexico; ^6^División de Neurociencias, Centro de Investigación Biomédica de Occidente, Instituto Mexicano del Seguro Social, Guadalajara, JAL, Mexico; ^7^Departamento de Clínicas Médicas, Centro Universitario de Ciencias de la Salud, Universidad de Guadalajara, Guadalajara, JAL, Mexico; ^8^Laboratorio de Mutagénesis, Centro de Investigación Biomédica de Occidente, Instituto Mexicano del Seguro Social, Guadalajara, JAL, Mexico

## Abstract

Genotoxic exposure to chemical substances is common, and nursing mothers could transmit harmful substances or their metabolites to their offspring through breast milk. We explored the possibility of determining genotoxic effects in the erythrocytes of breastfeeding rat pups whose mothers received a genotoxic compound while nursing. Ten groups of female rats and five pups per dam were studied. The control group received sterile water, and the experimental groups received one of three different doses of cyclophosphamide, colchicine, or cytosine-arabinoside. Blood smears were prepared from samples taken from each dam and pup every 24 h for six days. There were increased numbers of micronucleated erythrocytes (MNEs) and micronucleated polychromatic erythrocytes (MNPCEs) in the samples from pups in the experimental groups (*P* < 0.02) and increased MNPCE frequencies in the samples from the dams (*P* < 0.05). These results demonstrate the vertical transmission of the genotoxic effect of the compounds tested. In conclusion, assessing MNEs in breastfeeding neonate rats to assess DNA damage may be a useful approach for identifying genotoxic compounds and/or cytotoxic effects. This strategy could help in screening for therapeutic approaches that are genotoxic during the lactation stage and these assessments might also be helpful for developing preventive strategies to counteract harmful effects.

## 1. Introduction

The genetic integrity of the human population has been compromised by exposure to genotoxic agents. Several factors, such as lifestyle and medical treatments, could influence or induce damage to genetic material. Genotoxicity testing is necessary routine screening in populations that may be at risk to exposure [1]. Newborns can be exposed to drugs or other genotoxic agents, particularly through breast milk, and they are prime candidates for such testing approaches [2, 3].

Breast milk is the main source of nourishment for newborns [4–6]. The physiological, immunological, and neurological benefits of breast milk are widely documented [6–8]; however, many chemicals in the environment and various drugs have been identified as potential causes of cancer or birth defects. Other substances need to be recognized or tested for their teratogenic potential in humans during the perinatal period [9]. Since breast milk is the first way through which newborns may be exposed to toxins, it is a very important factor to consider.

Recent studies have shown that breastfeeding is increasing among mothers [7], but the suspension of breastfeeding is frequent, mainly due to therapeutic drugs prescribed by obstetricians [3, 6, 7, 10, 11]. Available recommendations for the use of drugs in breastfeeding women are based on the feasibility of the drug passing into breast milk [3, 6, 7, 12, 13].

The anti-inflammatory agent colchicine (COL) and the chemotherapeutic compounds cyclophosphamide (CP) and cytosine-arabinoside (AraC) are known micronucleogenic compounds that can be transferred through breast milk to infants. CP and AraC have been reported to promote prematurity, growth retardation, and mild abnormalities in transaminases. Although the transfer of COL through breast milk is not yet fully defined, it has been described to occur, and there is risk warning against using this compound during lactation [2, 3, 6].

Human milk extracts have been reported to cause DNA damage, which has also been observed in exfoliated cells recovered from the milk of women exposed to genotoxic agents [14–16]. This information also provides parameters to be considered as initiators of breast cancer. However, these studies did not assess the* in vivo* genotoxic potential in lactating neonates [17]. One way to explore this possibility is to perform the micronucleus (MN) test [18], which would show whether compounds administered to the mother could be transferred through the milk during lactation and have genotoxic effects in the offspring. In this study, we used neonatal rats as they are highly sensitive to genotoxicity assays [17]. Therefore, the present study aimed to identify whether it is possible to apply the rodent MN test to peripheral blood erythrocytes of nursing pups to observe the genotoxic and/or cytotoxic effects of three doses of three different compounds (CP, COL, and AraC) administered to their lactating mothers.

## 2. Methods

### 2.1. Animals

Fifty (50) female Wistar rats were provided by the Laboratory Animal Facility from the* Centro de Investigación Biomédica de Occidente, Instituto Mexicano del Seguro Social*. The rats were individually housed in polycarbonate cages and kept in laboratory conditions with access to food and water* ad libitum*. In addition, 250 pups of undetermined sex born from those rats (five per rat) were used during the experiments.

Female adult rats were mated, and pregnancy was established by the presence of sperm in a vaginal smear [19, 20]. The pups' day of birth was calculated once pregnancy was confirmed. After delivery, five newborn pups per mother were randomly selected for homogeneity among the groups and to assure appropriate offspring feeding [21, 22]; a total of 25 pups per group were analyzed.

### 2.2. MN Induction and Working Groups

MN induction in the experimental groups was performed by the administration of CP via oral gavage (CAS number 6055-19-2, Sigma-Aldrich) [17, 23], COL via oral gavage (CAS number 64-86-8, Sigma-Aldrich), or AraC by intramuscular administration (CAS number 147-94-4, Sigma-Aldrich) once daily for three days. These compounds are known to pass into breast milk [10, 24], although the transference of COL through breast milk is undefined. The following groups were formed (five rats per group), each containing a mother and five of her offspring: Group 1 (negative control), which was given sterile water; Groups 2, 3, and 4, which were given CP at 5, 10, or 20 mg/kg/day [17]; Groups 5, 6, and 7, which were given COL at 0.1, 0.2, or 0.4 mg/kg/day [25]; and Groups 8, 9, and 10, which were given AraC at 3, 4.5, or 6 mg/kg/day [26, 27], respectively. All doses administered by oral gavage were adjusted to a final volume of 0.5 mL with sterile water, and the doses administered by intramuscular administration were adjusted to a final volume of 0.2 mL with sterile water.

### 2.3. Sample Preparation and MNE Analysis

Six blood samples were collected from each rat (including the dam and pups) during the experiment. One drop of peripheral blood was collected once daily from the tip of the tail of each animal. The basal sample was taken immediately after delivery and prior to the administration of the compounds (0 h). Subsequent samples were taken daily over the next five days at 24, 48, 72, 96, and 120 h. Two smears were prepared from each sample time on precleaned and precoded microscope slides. The smears were air-dried, fixed in absolute ethanol for 10 min, and stained with acridine orange [25]. The slides were scored manually using an Olympus BX51 microscope equipped with epifluorescence and a 100x objective by two readers who were blinded to the sample data. For the adult rats, the number of micronucleated polychromatic erythrocytes (MNPCEs) was counted from 3,000 polychromatic erythrocytes (PCEs), and the proportion of PCEs was determined in 1,000 total erythrocytes (TEs; normochromatic and polychromatic erythrocytes). For the pups, the number of MNEs was determined in 10,000 TEs, and the number of MNPCEs was determined in 1,000 PCEs. The number of PCEs in 1,000 TEs was also counted as a system control because a decrease in PCE frequency reflects bone marrow toxicity of the drug and could lead to a misinterpretation of the MNE and MNPCE frequencies.

### 2.4. Ethical Considerations

This study was approved by the Institutional Research Committee and by a local Animal Care Committee. All experiments were performed in accordance with the guidelines for the care and use of experimental animals at the* Centro de Investigación Biomédica de Occidente*, which are in compliance with those given by national (México; NOM-062-ZOO-2001) and international guidelines for the humane treatment of research animals [28–31].

### 2.5. Statistical Analysis

All results (‰) are expressed as the mean ± standard deviation. The results were evaluated using the Statistical Program for Social Sciences (SPSS v. 18.0, IBM Co., Armonk, NY, USA). Distribution normality was determined using the Kolmogorov–Smirnoff test. The dams were used as the experimental unit (*n* = 5/group). A descriptive analysis was conducted for the variables. The MNPCE, MNE, and PCE frequencies were evaluated by the analysis of variance (ANOVA) of repeated measures and the Bonferroni* post hoc* test for multiple comparisons was applied for intragroup comparisons. For intergroup comparisons, one-way ANOVA was used, and Dunnett's test was employed to correct the significance values for multiple* post hoc* comparisons. PCE frequencies were evaluated by Student's* t*-test. *P* values <0.05 were considered statistically significant.

## 3. Results

All groups in this study were homogeneous, with similar average weights in the adults and rat pups at birth, regardless of the number of offspring per birth.

The average basal values (‰) obtained for the analyzed parameters in the peripheral blood derived from the dams were 1.28 ± 0.76 MNPCEs and 81.4 ± 26.1 PCEs, whereas the baseline values from all pups were 2.39 ± 0.7 MNEs, 3.61 ± 1.7 MNPCEs, and 603.3 ± 137.7 PCEs.

In samples taken from adult rats, there were significant differences (*P* < 0.05) in the MNPCE values (Table 1) in the groups exposed to any of the three compounds compared with the baseline values. These differences were not observed in the negative control group. Additionally, the highest CP dose led to differences in the PCE values (Table 2) after 72 h (*P* < 0.05), as determined by the intra- and intergroup comparisons.

Regarding the intergroup analysis, we found significant differences in the MNPCE values from dams compared with the corresponding negative control groups. These differences arose after 24 h in the dams from the lowest and higher dose of CP groups and in those from the middle- and higher-dose AraC groups.

In the intragroup analysis from adult rats, the MNPCE values increased significantly in the groups exposed to the three doses tested after 24 h in the COL and AraC groups and in those exposed to the lowest and highest doses of CP. In the middle-dose CP group, the increase in MNPCEs was observed after 48 h, and there were no differences in the negative control group for any of the sampling times (Table 1).

Data from the offspring of the experimental groups showed increased MNE (*P* < 0.01; Table 3) and MNPCE (*P* < 0.05; Table 4) and decreased PCE values (*P* < 0.01; Table 5), whereas the negative control group showed no differences in the MNE and MNPCE values for any of the samples.

In samples taken from pups, those in the low-dose CP group showed a significant difference (*P* < 0.002) in MNEs after 24 h, while differences were observed after 48 h in those in the middle- and higher-dose CP groups. This increase in MNEs was maintained for all subsequent sampling times (Table 3). In those in the COL groups, increased MNE frequencies were observed after 24 h for all 3 doses tested (*P* < 0.01). Finally, in those in the AraC groups, increased MNE frequencies (*P* < 0.02) were observed from 24 to 72 h for the lowest dose, from 24 h to 96 for the middle dose, and from 24 to 120 h for the highest dose (Table 3).

We also analyzed the number of MNPCEs per group at different times. We observed significant increases in the samples from pups in the CP and AraC groups after 24 h (*P* < 0.02), whereas increased MNPCEs in the samples from pups in the COL group were observed after 48 h (*P* < 0.02). In the negative control group, the values did not change during the sampling time (Table 4).

Pups in the highest-dose CP group showed the greatest increase at 72 h (*P* < 0.001), which was nearly 3 times the baseline value (Table 4), whereas in the highest-dose COL group the greatest increase was observed at 96 h. This group presented damage almost twice that of the baseline level (Table 4). Pups in the low-dose AraC group showed the higher increase in MNPCE values at 48 h, which was nearly four times higher than the baseline value (Table 4).

The intragroup comparisons showed a significant difference in the uniform decrease in PCEs in the pup samples from all groups (Table 5) (*P* < 0.003). However, the intergroup analysis, which compared the values over time with the corresponding value in the negative control group, revealed significant decrement from PCE values in the low-dose AraC group at 24 h, whereas the highest-dose COL group at 96 h and middle-dose AraC group at 120 h show increase in PCE values.

## 4. Discussion

It is entirely feasible that genotoxic compounds will inadvertently reach vulnerable populations, such as newborns. Since they might be receiving such agents through breast milk [3, 10, 24, 32], it is important to identify genotoxic compounds that can be transmitted via this route. In this study, three compounds were tested at three different doses to verify whether it was possible to detect genotoxic effects in nursing offspring.

Studies of compounds or metabolites transmitted via breastfeeding are currently based on direct measurements of the agents in milk. For this study, it was necessary to know in advance which methods and equipment are appropriate for evaluating each chemical compound. However, assessing the effects of these compounds in nursing pups is difficult and typically involves complex technical methods [6, 7, 15].

Adult rats have a very low number of MNEs in their peripheral blood [33] due to the efficiency of the spleen in withdrawing them from circulation [34, 35]. However, it has been reported that newborn rats have significantly higher MN numbers [17, 33].

The present study conducted using neonatal rats is justified since they present MNEs in peripheral blood [17, 33]. The analysis used an MN test to assess vertical transmission through breast milk. The aim was to detect damage to genetic material that could occur via the transfer of the analyzed compounds through the mother by breastfeeding.

The three doses of the three agents tested here resulted in increased MNEs and MNPCEs in the pup samples. When the genotoxic agents were administered to the dams during the lactation period, blood samples were collected from them to assess the effects of the genotoxic agents and to assure that the doses were adequate to cause an MNE increase in the mothers, corroborating the effects of these compounds. As shown in Tables 1, 3, and 4, the expected damage caused by CP, COL, and AraC was observed in all cases.

Although the weights of the pups at birth were not significantly different, pups in the low-dose CP group showed a slightly lower basal weight, which could be one of the reasons why this group showed higher CP genotoxicity values (Table 3): smaller organisms may be more immature and thus more susceptible to damage. Additionally, this result may also have been because there were more pups per litter. Furthermore, the dosing method could affect the results. The CP was administered to the mother in doses per kilogram of body weight. In the low-dose group, the dose received by each pup could be higher than in the other groups because of their smaller size, which would explain their greater sensitivity to the effects of the drug.

In the intragroup comparison of MNPCE frequencies, there were no differences in the values from offspring in the low- and middle-dose CP groups at 24 h relative to the baseline measurements, whereas the other groups showed differences after 24 h from the first administration of the compounds. This finding suggests higher levels of recent DNA damage in those groups. The genotoxic effects were also observed in the MNPCE values from the dams.

The ratio of PCEs/TEs is used as a control system that helps in determining a compound's cytotoxicity due to its myelosuppressive effect. Table 2 shows that no significant changes occurred in the PCE values in the dams exposed to COL or AraC at the three doses tested, which is evidence of genotoxicity without cytotoxicity; however, in the CP groups, differences were observed with the highest dose at 72 and 96 h.

Additionally, lower PCE values in the offspring were uniform in all the experimental groups and the negative control group (Table 5). However, the intergroup analysis revealed that the decrease was smaller in the control group than in the experimental groups and was thus discarded as potential cytotoxic effect of the genotoxic agent. These decreases in frequencies appear to be normal in offspring maturing over time [20, 36]. Once a pup is born, it no longer needs to circulate a larger number of immature cells because the amount of available oxygen changes [37, 38]. As the individual matures, it will achieve a balance in the production of young cells. Thus, the number of PCEs, which are initially in greater quantity in circulation, is modified. The number of PCEs diminishes and those of normochromatic or mature red blood cells in peripheral circulation increase, as observed in mature animals. These approaches explain the reduced PCE values in the different groups. Although the intragroup analysis showed significant differences in PCE values, the intergroup analysis showed that the experimental groups had the same behavior as the control group. Therefore, due to the PCE decrease observed in pups from all groups, we can discard the cytotoxic effect of the three doses administered from the three compounds tested.

MN increases were observed in the experimental groups but in a non-dose-dependent manner. This result could be because the quantity of metabolites transmitted was too low to observe drastic effects. In the present study, the doses of CP, COL, and AraC were administered to the mother in the breastfeeding period, meaning that the offspring were indirectly exposed. Thus, to exert its genotoxic effect, the compound had to pass first through the maternal metabolic pathways before passing into the maternal milk; then, to affect the pups, the compound must pass directly into the milk and the pup without first being metabolized by the mother, the pup, or both. Another point to consider is that breastfeeding is known to protect newborns from deleterious effects, such as oxidative stress, drug exposure, and infections, which can alter genetic material, especially during vulnerable life stages [38]. In this sense, it is possible that the chosen doses could be too similar and that more distinct doses would show different effects due to the more obvious intervals between each dose.

Having found statistically significant differences in the MN values of the experimental groups, we demonstrated that CP, COL, and AraC could be suitable as positive controls for this type of analysis. In addition, the lowest CP dose (5 mg/kg) is suitable as a positive control for evaluating the possible genotoxic effects of other compounds. The standardized CP dose typically used as a positive control in rodents is a single dose of 50 mg/kg [39], but in this work, a total of 25 mg/kg was administered over the 5-day experimental period, with a good response.

In the case of CP and AraC, both compounds can be transferred through breast milk to pups, and although COL has been described to be capable of transferring through breast milk, it is not yet clearly defined. As such, there should be a risk warning against using this compound during the lactation period. Based on the genotoxic effect observed in the offspring in this study, we can suggest that COL can exert an effect through milk.

This rat model showed sufficiently sensitive response to the lower doses, thus presenting the advantage of using young animal models. In this case, newborn animals were more susceptible, and the effects of the damage were very clear [17, 19, 20, 33, 40].

Neonates of several animal species have been reported to show high MNE frequencies [33]. These species could be alternatives for assessing genotoxicity transmitted through milk. Animal models are advantageous because they enable compounds to be metabolized into others that might be more or less toxic than the original compound. If drugs can cause damage of DNA in laboratory animals, similar effects could potentially occur in humans. At this point, it is important to mention that the presence of MNEs in premature human infants is similar to the behavior observed in rats and other species [33], which also have relatively high numbers of MNEs in the peripheral blood [33]. As time passes and pups mature, the control over these structures becomes more efficient.

It has been reported that infants can be exposed to genotoxic compounds through breastfeeding because genotoxic compounds can accumulate in fatty tissue; these compounds may also be initiators of breast cancer. It is extremely important to know whether chemical compounds to which mothers are exposed would damage their own DNA and/or that of their offspring [14–16].

In conclusion, in the present study, we observed the genotoxic effects of CP, COL, and AraC in the peripheral blood of rat pups. The pups were exposed to the compounds via breastfeeding from treated mothers. Applying the MN test to the peripheral blood erythrocytes of newborn rats exposed by lactation to substances suspected to have a micronucleogenic potential may be useful for identifying genotoxic compounds that could be harmful to the pups. This approach may be a simple means of determining whether a micronucleogenic compound may pass through breast milk to offspring. In addition, this approach could facilitate the establishment of additional therapeutic strategies and informed guidelines for the use or restriction of drugs during the breastfeeding period and aid in the discovery of preventive measures and strategies to counteract such effects.

## Figures and Tables

**Table 1:** MNPCE frequencies from dams of rat study groups at different sampling times.

Groups	0 h	24 h	48 h	72 h	96 h	120 h
Negative control	1.40 ± 0.89	1.80 ± 1.30	1.80 ± 0.84	1.60 ± 1.14	2.00 ± 1.30	2.20 ± 1.30
		^*∗*^NS	^*∗*^NS	^*∗*^NS	^*∗*^NS	^*∗*^NS

CP lower-dose	1.20 ± 0.83 ^*∗∗*^NS	4.47 ± 1.45	13.47 ± 3.53	12.07 ± 3.61	7.33 ± 3.48	2.53 ± 1.12
		^*∗*^ *P* = 0.024	^*∗*^ *P* = 0.003	^*∗*^ *P* = 0.005	^*∗*^ *P* = 0.029	^*∗*^NS
		^*∗∗*^ *P* = 0.043	^*∗∗*^ *P* = 0.006	^*∗∗*^ *P* < 0.001	^*∗∗*^NS	^*∗∗*^NS

CP middle-dose	1.60 ± 0.89 ^*∗∗*^NS	3.47 ± 1.61	20.60 ± 4.34	13.40 ± 3.58	14.07 ± 4.75	2.07 ± 0.86
		^*∗*^NS	^*∗*^ *P* < 0.001	^*∗*^ *P* = 0.002	^*∗*^ *P* = 0.007	^*∗*^NS
		^*∗∗*^NS	^*∗∗*^ *P* = 0.002	^*∗∗*^ *P* = 0.005	^*∗∗*^ *P* = 0.018	^*∗∗*^NS

CP higher-dose	1.27 ± 0.43 ^*∗∗*^NS	6.73 ± 2.12	24.33 ± 5.25	20.20 ± 6.21	16.80 ± 4.27	3.60 ± 1.38
		^*∗*^ *P* = 0.006	^*∗*^ *P* = 0.001	^*∗*^ *P* = 0.003	^*∗*^ *P* = 0.002	^*∗*^ *P* = 0.033
		^*∗∗*^ *P* = 0.003	^*∗∗*^ *P* = 0.002	^*∗∗*^ *P* < 0.001	^*∗∗*^ *P* = 0.005	^*∗∗*^NS

COL lower-dose	1.00 ± 0.71 ^*∗∗*^NS	3.00 ± 0.71	5.00 ± 1.87	7.60 ± 2.40	7.20 ± 1.64	3.00 ± 1.22
		^*∗*^ *P* = 0.011	^*∗*^ *P* = 0.025	^*∗*^ *P* = 0.007	^*∗*^ *P* = 0.003	^*∗*^ *P* = 0.022
		^*∗∗*^NS	^*∗∗*^NS	^*∗∗*^ *P* = 0.014	^*∗∗*^ *P* = 0.003	^*∗∗*^NS

COL middle-dose	1.20 ± 0.45 ^*∗∗*^NS	3.80 ± 1.79	7.00 ± 1.22	7.60 ± 2.51	6.00 ± 2.55	3.20 ± 0.84
		^*∗*^ *P* = 0.033	^*∗*^ *P* = 0.001	^*∗*^ *P* = 0.003	^*∗*^ *P* = 0.009	^*∗*^ *P* = 0.011
		^*∗∗*^NS	^*∗∗*^ *P* = 0.001	^*∗∗*^ *P* = 0.016	^*∗∗*^NS	^*∗∗*^NS

COL higher-dose	1.20 ± 0.84 ^*∗∗*^NS	3.60 ± 1.52	5.80 ± 1.92	6.40 ± 2.40	6.40 ± 2.41	3.60 ± 1.82
		^*∗*^ *P* = 0.024	^*∗*^ *P* = 0.006	^*∗*^ *P* = 0.014	^*∗*^ *P* = 0.012	^*∗*^NS
		^*∗∗*^NS	^*∗∗*^ *P* = 0.031	^*∗∗*^ *P* = 0.036	^*∗∗*^NS	^*∗∗*^NS

AraC lower-dose	1.20 ± 0.84 ^*∗∗*^NS	3.80 ± 1.79	9.20 ± 2.38	8.00 ± 2.74	3.60 ± 0.89	1.80 ± 0.45
		^*∗*^ *P* = 0.041	^*∗*^ *P* = 0.002	^*∗*^ *P* = 0.003	^*∗*^ *P* = 0.024	^*∗*^NS
		^*∗∗*^NS	^*∗∗*^ *P* = 0.006	^*∗∗*^ *P* = 0.009	^*∗∗*^NS	^*∗∗*^NS

AraC middle-dose	1.20 ± 1.10 ^*∗∗*^NS	5.60 ± 1.82	8.80 ± 2.39	7.80 ± 1.30	5.80 ± 1.30	3.40 ± 1.52
		^*∗*^ *P* = 0.022	^*∗*^ *P* = 0.006	^*∗*^ *P* = 0.003	^*∗*^ *P* = 0.006	^*∗*^NS
		^*∗∗*^ *P* = 0.013	^*∗∗*^ *P* = 0.008	^*∗∗*^ *P* < 0.001	^*∗∗*^ *P* = 0.006	^*∗∗*^NS

AraC higher-dose	1.40 ± 0.89 ^*∗∗*^NS	7.40 ± 2.07	9.00 ± 2.00	8.20 ± 2.49	8.00 ± 2.00	3.40 ± 1.51
		^*∗*^ *P* = 0.001	^*∗*^ *P* = 0.002	^*∗*^ *P* = 0.003	^*∗*^ *P* = 0.001	^*∗*^ *P* = 0.047
		^*∗∗*^ *P* = 0.001	^*∗∗*^ *P* = 0.003	^*∗∗*^ *P* = 0.010	^*∗∗*^ *P* = 0.005	^*∗∗*^NS

Data (‰) expressed as mean ± standard deviation per group of MNPCEs/1,000 PCEs. MNPCEs: micronucleated polychromatic erythrocytes, PCEs: polychromatic erythrocytes, h: hours, and NS: not significant. ^*∗*^Intragroup comparisons. ^*∗∗*^Intergroup comparisons between negative control groups versus the other groups.

**Table 2:** PCE frequencies from dams of rat study groups at the different sampling times.

Groups	0 h	24 h	48 h	72 h	96 h	120 h
Negative control	78.00 ± 27.17	84.20 ± 29.77	79.60 ± 25.40	88.00 ± 23.39	81.80 ± 23.42	89.40 ± 22.43
		^*∗*^NS	^*∗*^NS	^*∗*^NS	^*∗*^NS	^*∗*^NS

CP lower-dose	83.20 ± 20.33 ^*∗∗*^NS	82.20 ± 53.89	82.20 ± 45.06	83.40 ± 46.95	80.60 ± 30.66	86.60 ± 46.12
		^*∗*^NS	^*∗*^NS	^*∗*^NS	^*∗*^NS	^*∗*^NS
		^*∗∗*^NS	^*∗∗*^NS	^*∗∗*^NS	^*∗∗*^NS	^*∗∗*^NS

CP middle-dose	74.60 ± 23.33 ^*∗∗*^NS	84.40 ± 37.66	78.80 ± 58.31	68.80 ± 41.15	76.20 ± 23.84	83.40 ± 30.30
		^*∗*^NS	^*∗*^NS	^*∗*^NS	^*∗*^NS	^*∗*^NS
		^*∗∗*^NS	^*∗∗*^NS	^*∗∗*^NS	^*∗∗*^NS	^*∗∗*^NS

CP higher-dose	77.40 ± 10.71 ^*∗∗*^NS	56.40 ± 19.83	48.80 ± 19.86	34.40 ± 16.41	30.20 ± 17.02	44.20 ± 16.97
		^*∗*^NS	^*∗*^NS	^*∗*^ *P* = 0.037	^*∗*^ *P* = 0.047	^*∗*^NS
		^*∗∗*^NS	^*∗∗*^NS	^*∗∗*^ *P* = 0.003	^*∗∗*^ *P* = 0.004	^*∗∗*^ *P* = 0.007

COL lower-dose	90.80 ± 44.42 ^*∗∗*^NS	100.00 ± 60.46	92.00 ± 45.91	93.20 ± 43.89	99.00 ± 49.40	97.20 ± 57.93
		^*∗*^NS	^*∗*^NS	^*∗*^NS	^*∗*^NS	^*∗*^NS
		^*∗∗*^NS	^*∗∗*^NS	^*∗∗*^NS	^*∗∗*^NS	^*∗∗*^NS

COL middle-dose	87.20 ± 42.71 ^*∗∗*^NS	97.40 ± 52.79	105.60 ± 54.24	129.00 ± 38.40	106.00 ± 37.28	102.40 ± 60.16
		^*∗*^NS	^*∗*^NS	^*∗*^NS	^*∗*^NS	^*∗*^NS
		^*∗∗*^NS	^*∗∗*^NS	^*∗∗*^NS	^*∗∗*^NS	^*∗∗*^NS

COL higher-dose	81.80 ± 28.09 ^*∗∗*^NS	99.60 ± 63.70	89.40 ± 38.37	131.40 ± 61.67	119.40 ± 43.84	97.00 ± 39.86
		^*∗*^NS	^*∗*^NS	^*∗*^NS	^*∗*^NS	^*∗*^NS
		^*∗∗*^NS	^*∗∗*^NS	^*∗∗*^NS	^*∗∗*^NS	^*∗∗*^NS

AraC lower-dose	83.40 ± 23.44 ^*∗∗*^NS	95.80 ± 56.98	99.60 ± 52.55	94.80 ± 60.72	105.00 ± 61.70	105.60 ± 76.51
		^*∗*^NS	^*∗*^NS	^*∗*^NS	^*∗*^NS	^*∗*^NS
		^*∗∗*^NS	^*∗∗*^NS	^*∗∗*^NS	^*∗∗*^NS	^*∗∗*^NS

AraC middle-dose	82.60 ± 24.78 ^*∗∗*^NS	82.20 ± 28.38	103.40 ± 65.58	106.60 ± 62.77	95.80 ± 41.43	102.40 ± 38.41
		^*∗*^NS	^*∗*^NS	^*∗*^NS	^*∗*^NS	^*∗*^NS
		^*∗∗*^NS	^*∗∗*^NS	^*∗∗*^NS	^*∗∗*^NS	^*∗∗*^NS

AraC higher-dose	82.20 ± 26.48 ^*∗∗*^NS	86.00 ± 49.10	95.00 ± 35.62	83.80 ± 34.60	98.80 ± 51.84	89.60 ± 22.22
		^*∗*^NS	^*∗*^NS	^*∗*^NS	^*∗*^NS	^*∗*^NS
		^*∗∗*^NS	^*∗∗*^NS	^*∗∗*^NS	^*∗∗*^NS	^*∗∗*^NS

Data (‰) expressed as mean ± standard deviation per group of PCEs/1,000 TEs. PCEs: polychromatic erythrocytes, TEs: total erythrocytes, h: hours, and NS: not significant. ^*∗*^Intragroup comparisons. ^*∗∗*^Intergroup comparisons between negative control groups versus the other groups.

**Table 3:** MNE frequencies from pup samples of rat study groups at different sampling times.

Groups	0 h	24 h	48 h	72 h	96 h	120 h
Negative control	2.25 ± 0.6	2.34 ± 0.5	2.48 ± 0.7	2.37 ± 0.6	2.55 ± 0.6	2.51 ± 0.7
		^*∗*^NS	^*∗*^NS	^*∗*^NS	^*∗*^NS	^*∗*^NS

CP lower-dose	2.21 ± 0.3 ^*∗∗*^NS	2.77 ± 0.6	3.76 ± 1.1	4.62 ± 1.0	4.51 ± 1.0	4.11 ± 1.0
		^*∗*^ *P* < 0.001	^*∗*^ *P* < 0.001	^*∗*^ *P* < 0.001	^*∗*^ *P* < 0.001	^*∗*^ *P* < 0.001
		^*∗∗*^NS	^*∗∗*^ *P* = 0.001	^*∗∗*^ *P* < 0.001	^*∗∗*^ *P* < 0.001	^*∗∗*^ *P* < 0.001

CP middle-dose	2.13 ± 0.8 ^*∗∗*^NS	2.47 ± 0.8	3.27 ± 0.9	3.87 ± 1.2	3.81 ± 1.1	3.60 ± 1.2
		^*∗*^NS	^*∗*^ *P* = 0.001	^*∗*^ *P* < 0.001	^*∗*^ *P* < 0.001	^*∗*^ *P* < 0.001
		^*∗∗*^NS	^*∗∗*^NS	^*∗∗*^ *P* < 0.001	^*∗∗*^ *P* < 0.001	^*∗∗*^ *P* < 0.008

CP higher-dose	2.46 ± 0.6 ^*∗∗*^NS	2.68 ± 0.9	3.65 ± 0.9	4.07 ± 1.0	3.97 ± 0.8	4.08 ± 1.2
		^*∗*^NS	^*∗*^ *P* < 0.001	^*∗*^ *P* < 0.001	^*∗*^ *P* < 0.001	^*∗*^ *P* < 0.001
		^*∗∗*^NS	^*∗∗*^ *P* < 0.001	^*∗∗*^ *P* < 0.001	^*∗∗*^ *P* < 0.001	^*∗∗*^ *P* < 0.001

COL lower-dose	2.68 ± 0.6 ^*∗∗*^NS	3.24 ± 1.0	3.58 ± 1.1	4.13 ± 1.2	4.17 ± 1.0	4.07 ± 1.1
		^*∗*^ *P* = 0.008	^*∗*^ *P* < 0.002	^*∗*^ *P* < 0.001	^*∗*^ *P* < 0.001	^*∗*^ *P* < 0.001
		^*∗∗*^ *P* = 0.01	^*∗∗*^ *P* = 0.006	^*∗∗*^ *P* < 0.001	^*∗∗*^ *P* < 0.001	^*∗∗*^ *P* < 0.001

COL middle-dose	2.41 ± 0.8 ^*∗∗*^NS	3.03 ± 0.8	3.62 ± 1.2	3.80 ± 0.9	3.96 ± 1.5	4.27 ± 1.2
		^*∗*^ *P* < 0.003	^*∗*^ *P* < 0.001	^*∗*^ *P* < 0.001	^*∗*^ *P* < 0.001	^*∗*^ *P* < 0.001
		^*∗∗*^ *P* = 0.015	^*∗∗*^ *P* = 0.003	^*∗∗*^ *P* < 0.001	^*∗∗*^ *P* = 0.004	^*∗∗*^ *P* < 0.001

COL higher-dose	2.75 ± 0.8 ^*∗∗*^NS	4.13 ± 1.1	4.77 ± 1.2	4.40 ± 1.5	5.00 ± 1.4	5.09 ± 1.5
		^*∗*^ *P* < 0.001	^*∗*^ *P* < 0.001	^*∗*^ *P* < 0.001	^*∗*^ *P* < 0.001	^*∗*^ *P* < 0.001
		^*∗∗*^ *P* < 0.001	^*∗∗*^ *P* < 0.001	^*∗∗*^ *P* < 0.001	^*∗∗*^ *P* < 0.001	^*∗∗*^ *P* < 0.001

AraC lower-dose	2.20 ± 1.2 ^*∗∗*^NS	2.93 ± 1.5	3.45 ± 1.1	3.25 ± 1.3	2.78 ± 1.5	2.24 ± 1.1
		^*∗*^ *P* = 0.080	^*∗*^ *P* = 0.008	^*∗*^ *P* = 0.017	^*∗*^NS	^*∗*^NS
		^*∗∗*^NS	^*∗∗*^ *P* = 0.02	^*∗∗*^ *P* = 0.046	^*∗∗*^NS	^*∗∗*^NS

AraC middle-dose	2.62 ± 1.2 ^*∗∗*^NS	3.40 ± 1.5	3.75 ± 1.1	3.78 ± 1.5	3.28 ± 1.8	3.23 ± 1.6
		^*∗*^ *P* = 0.007	^*∗*^ *P* = 0.002	^*∗*^ *P* = 0.006	^*∗*^ *P* = 0.036	^*∗*^NS
		^*∗∗*^ *P* = 0.029	^*∗∗*^ *P* = 0.01	^*∗∗*^ *P* = 0.003	^*∗∗*^NS	^*∗∗*^NS

AraC higher-dose	2.55 ± 0.6 ^*∗∗*^NS	4.14 ± 1.9	3.72 ± 1.5	3.36 ± 0.9	3.33 ± 1.0	3.44 ± 1.6
		^*∗*^ *P* < 0.001	^*∗*^ *P* = 0.004	^*∗*^ *P* < 0.001	^*∗*^ *P* = 0.005	^*∗*^ *P* < 0.041
		^*∗∗*^ *P* = 0.003	^*∗∗*^ *P* = 0.012	^*∗∗*^ *P* = 0.001	^*∗∗*^ *P* = 0.023	^*∗∗*^NS

Data (‰) expressed as mean ± standard deviation per group of MNEs/1,000 TEs. MNEs: micronucleated erythrocytes, TEs: total erythrocytes, h: hours, and NS: not significant. ^*∗*^Intragroup comparisons. ^*∗∗*^Intergroup comparisons between negative control groups versus the other groups.

**Table 4:** MNPCE frequencies from pup samples of rat study groups at different sampling times.

Groups	0 h	24 h	48 h	72 h	96 h	120 h
Negative control	3.75 ± 1.6	3.80 ± 1.6	4.15 ± 1.8	4.30 ± 1.9	4.30 ± 1.4	4.25 ± 1.4
		^*∗*^NS	^*∗*^NS	^*∗*^NS	^*∗*^NS	^*∗*^NS

CP lower-dose	3.75 ± 1.8 ^*∗∗*^NS	5.00 ± 2.4	6.80 ± 2.1	7.75 ± 2.3	7.75 ± 2.1	7.45 ± 2.2
		^*∗*^ *P* = 0.015	^*∗*^ *P* < 0.001	^*∗*^ *P* < 0.001	^*∗*^ *P* < 0.001	^*∗*^ *P* < 0.001
		^*∗∗*^NS	^*∗∗*^ *P* = 0.001	^*∗∗*^ *P* < 0.001	^*∗∗*^ *P* < 0.001	^*∗∗*^ *P* < 0.001

CP middle-dose	3.00 ± 1.3 ^*∗∗*^NS	4.80 ± 1.8	6.70 ± 2.5	7.75 ± 3.5	9.30 ± 4.3	7.65 ± 3.9
		^*∗*^ *P* = 0.006	^*∗*^ *P* < 0.001	^*∗*^ *P* < 0.001	^*∗*^ *P* < 0.001	^*∗*^ *P* < 0.001
		^*∗∗*^NS	^*∗∗*^ *P* = 0.005	^*∗∗*^ *P* = 0.004	^*∗∗*^ *P* < 0.001	^*∗∗*^ *P* = 0.009

CP higher-dose	3.60 ± 1.5 ^*∗∗*^NS	5.35 ± 2.3	7.40 ± 3.7	9.70 ± 4.2	8.70 ± 3.3	7.00 ± 3.4
		^*∗*^ *P* = 0.003	^*∗*^ *P* < 0.001	^*∗*^ *P* < 0.001	^*∗*^ *P* < 0.001	^*∗*^ *P* < 0.001
		^*∗∗*^NS	^*∗∗*^ *P* = 0.002	^*∗∗*^ *P* < 0.001	^*∗∗*^ *P* = 0.015	^*∗∗*^ *P* = 0.015

COL lower-dose	4.45 ± 1.4 ^*∗∗*^NS	4.80 ± 2.0	6.65 ± 2.9	7.15 ± 2.7	6.90 ± 2.4	7.25 ± 2.9
		^*∗*^NS	^*∗*^ *P* < 0.001	^*∗*^ *P* < 0.002	^*∗*^ *P* = 0.001	^*∗*^ *P* = 0.001
		^*∗∗*^NS	^*∗∗*^ *P* = 0.016	^*∗∗*^ *P* = 0.003	^*∗∗*^ *P* = 0.002	^*∗∗*^ *P* = 0.002

COL middle-dose	4.60 ± 1.9 ^*∗∗*^NS	5.40 ± 2.5	7.40 ± 3.9	6.85 ± 3.3	7.10 ± 2.2	6.75 ± 3.4
		^*∗*^NS	^*∗*^ *P* = 0.002	^*∗*^ *P* = 0.001	^*∗*^ *P* < 0.001	^*∗*^ *P* = 0.017
		^*∗∗*^ *P* = 0.045	^*∗∗*^ *P* < 0.013	^*∗∗*^NS	^*∗∗*^ *P* < 0.001	^*∗∗*^NS

COL higher-dose	4.80 ± 1.6 ^*∗∗*^NS	6.70 ± 2.7	7.50 ± 2.0	6.70 ± 1.9	7.95 ± 3.2	7.25 ± 2.6
		^*∗*^NS	^*∗*^ *P* = 0.003	^*∗*^ *P* < 0.001	^*∗*^ *P* = 0.001	^*∗*^ *P* = 0.001
		^*∗∗*^ *P* = 0.004	^*∗∗*^ *P* = 0.001	^*∗∗*^ *P* = 0.002	^*∗∗*^ *P* = 0.001	^*∗∗*^ *P* = 0.001

AraC lower-dose	2.50 ± 1.2 ^*∗∗*^NS	5.1 ± 2.7	9.60 ± 5.6	7.10 ± 5.7	6.25 ± 2.9	2.95 ± 1.4
		^*∗*^ *P* < 0.001	^*∗*^ *P* < 0.001	^*∗*^ *P* < 0.001	^*∗*^ *P* = 0.002	^*∗*^NS
		^*∗∗*^NS	^*∗∗*^ *P* = 0.002	^*∗∗*^NS	^*∗∗*^NS	^*∗∗*^ *P* = 0.033

AraC middle-dose	2.65 ± 1.2 ^*∗∗*^NS	4.55 ± 1.3	7.95 ± 3.3	7.65 ± 3.2	5.30 ± 2.4	5.15 ± 2.5
		^*∗*^ *P* < 0.001	^*∗*^ *P* < 0.001	^*∗*^ *P* < 0.001	^*∗*^ *P* < 0.001	^*∗*^ *P* < 0.001
		^*∗∗*^NS	^*∗∗*^ *P* = 0.001	^*∗∗*^ *P* = 0.005	^*∗∗*^NS	^*∗∗*^NS

AraC higher-dose	2.75 ± 1.4 ^*∗∗*^NS	7.10 ± 3.4	8.75 ± 3.0	6.45 ± 1.8	5.25 ± 2.3	4.00 ± 2.6
		^*∗*^ *P* < 0.001	^*∗*^ *P* < 0.001	^*∗*^ *P* = 0.001	^*∗*^ *P* < 0.001	^*∗*^NS
		^*∗∗*^ *P* = 0.001	^*∗∗*^ *P* < 0.001	^*∗∗*^ *P* = 0.004	^*∗∗*^NS	^*∗∗*^NS

Data (‰) expressed as mean ± standard deviation per group of MNPCEs/1,000 PCEs. MNPCEs: micronucleated polychromatic erythrocytes, PCEs: polychromatic erythrocytes, h: hours, and NS: not significant. ^*∗*^Intragroup comparisons. ^*∗∗*^Intergroup comparisons between negative control groups versus the other groups.

**Table 5:** PCE frequencies of pup samples of rat study groups at the different sampling times.

Groups	0 h	24 h	48 h	72 h	96 h	120 h
Negative control	608.8 ± 160.7	430.7 ± 86.8	373.9 ± 89.1	375.4 ± 100.5	389.0 ± 71.9	394.9 ± 67.2
		^*∗*^ *P* < 0.001	^*∗*^ *P* < 0.001	^*∗*^ *P* < 0.001	^*∗*^ *P* < 0.001	^*∗*^ *P* < 0.001

CP lower-dose	605.0 ± 138.7 ^*∗∗*^NS	432.7 ± 91.6	397.1 ± 68.1	413.7 ± 89.7	405.2 ± 81.5	401.3 ± 70.9
		^*∗*^ *P* < 0.001	^*∗*^ *P* < 0.001	^*∗*^ *P* < 0.001	^*∗*^ *P* < 0.001	^*∗*^ *P* < 0.001
		^*∗∗*^NS	^*∗∗*^NS	^*∗∗*^NS	^*∗∗*^NS	^*∗∗*^NS

CP middle-dose	618.8 ± 120.6 ^*∗∗*^NS	413.6 ± 94.4	338.7 ± 80.7	342.6 ± 53.6	362.5 ± 59.0	399.2 ± 82.1
		^*∗*^ *P* < 0.001	^*∗*^ *P* < 0.001	^*∗*^ *P* < 0.001	^*∗*^ *P* < 0.001	^*∗*^ *P* < 0.001
		^*∗∗*^NS	^*∗∗*^NS	^*∗∗*^NS	^*∗∗*^NS	^*∗∗*^NS

CP higher-dose	632.6 ± 138.0 ^*∗∗*^NS	457.0 ± 100.5	379.3 ± 80.6	352.3 ± 81.9	379.9 ± 55.3	388.7 ± 92.0
		^*∗*^ *P* < 0.001	^*∗*^ *P* < 0.001	^*∗*^ *P* < 0.001	^*∗*^ *P* < 0.001	^*∗*^ *P* < 0.001
		^*∗∗*^NS	^*∗∗*^NS	^*∗∗*^NS	^*∗∗*^NS	^*∗∗*^NS

COL lower-dose	621.1 ± 129.7 ^*∗∗*^NS	490.0 ± 95.5	391.5 ± 79.9	407.3 ± 63.1	415.3 ± 69.3	441.6 ± 66.4
		^*∗*^ *P* < 0.001	^*∗*^ *P* < 0.001	^*∗*^ *P* < 0.001	^*∗*^ *P* < 0.001	^*∗*^ *P* < 0.001
		^*∗∗*^NS	^*∗∗*^NS	^*∗∗*^NS	^*∗∗*^NS	^*∗∗*^NS

COL middle-dose	571.6 ± 138.2 ^*∗∗*^NS	468.6 ± 94.6	389.2 ± 56.2	380.7 ± 119.2	396.7 ± 54.1	419.8 ± 77.8
		^*∗*^ *P* = 0.001	^*∗*^ *P* < 0.001	^*∗*^ *P* = 0.001	^*∗*^ *P* < 0.001	^*∗*^ *P* < 0.001
		^*∗∗*^NS	^*∗∗*^NS	^*∗∗*^NS	^*∗∗*^NS	^*∗∗*^NS

COL higher-dose	667.2 ± 73.5 ^*∗∗*^NS	483.2 ± 114.5	392.5 ± 61.4	429.5 ± 58.6	457.5 ± 63.0	440.5 ± 65.2
		^*∗*^ *P* < 0.001	^*∗*^ *P* < 0.001	^*∗*^ *P* < 0.001	^*∗*^ *P* < 0.001	^*∗*^ *P* < 0.001
		^*∗∗*^NS	^*∗∗*^NS	^*∗∗*^NS	^*∗∗*^ *P* = 0.011	^*∗∗*^NS

AraC lower-dose	606.2 ± 133.8 ^*∗∗*^NS	344.8 ± 102.0	373.9 ± 72.0	391.9 ± 70.3	392.8 ± 61.8	433.5 ± 62.3
		^*∗*^ *P* < 0.001	^*∗*^ *P* < 0.001	^*∗*^ *P* < 0.001	^*∗*^ *P* < 0.001	^*∗*^ *P* < 0.001
		^*∗∗*^ *P* = 0.030	^*∗∗*^NS	^*∗∗*^NS	^*∗∗*^NS	^*∗∗*^NS

AraC middle-dose	555.8 ± 106.9 ^*∗∗*^NS	393.8 ± 89.4	327.2 ± 49.4	381.9 ± 87.8	439.6 ± 78.1	498.3 ± 81.1
		^*∗*^ *P* = 0.001	^*∗*^ *P* < 0.001	^*∗*^ *P* < 0.001	^*∗*^ *P* < 0.001	^*∗*^ *P* = 0.024
		^*∗∗*^NS	^*∗∗*^NS	^*∗∗*^NS	^*∗∗*^NS	^*∗∗*^ *P* < 0.001

AraC higher-dose	535.5 ± 153.7 ^*∗∗*^NS	404.8 ± 73.6	361.2 ± 65.0	335.2 ± 60.7	386.9 ± 77.2	424.9 ± 102.0
		^*∗*^ *P* = 0.001	^*∗*^ *P* < 0.001	^*∗*^ *P* < 0.001	^*∗*^ *P* = 0.004	^*∗*^ *P* = 0.030
		^*∗∗*^NS	^*∗∗*^NS	^*∗∗*^NS	^*∗∗*^NS	^*∗∗*^NS

Data (‰) expressed as mean ± standard deviation per group of PCEs/1,000 TEs. PCEs: polychromatic erythrocytes, TEs: total erythrocytes, h: hours, and NS: not significant. ^*∗*^Intragroup comparisons. ^*∗∗*^Intergroup comparisons between negative control groups versus the other groups.
